# Vascular Closure Devices versus Manual Compression in Cardiac Interventional Procedures: Systematic Review and Meta-Analysis

**DOI:** 10.1155/2022/8569188

**Published:** 2022-09-09

**Authors:** Naidong Pang, Jia Gao, Binghang Zhang, Min Guo, Nan Zhang, Meng Sun, Rui Wang

**Affiliations:** ^1^Department of Cardiology, First Hospital of Shanxi Medical University, Taiyuan, Shanxi, China; ^2^First Clinical Medical College, Shanxi Medical University, Taiyuan, Shanxi, China

## Abstract

**Backgrounds:**

Manual compression (MC) and vascular closure device (VCD) are two methods of vascular access site hemostasis after cardiac interventional procedures. However, there is still controversial over the use of them and a lack of comprehensive and systematic meta-analysis on this issue.

**Methods:**

Original articles comparing VCD and MC in cardiac interventional procedures were searched in PubMed, EMbase, Cochrane Library, and Web of Science through April 2022. Efficacy, safety, patient satisfaction, and other parameters were assessed between two groups. Heterogeneity among studies was evaluated by *I^2^* index and the Cochran *Q* test, respectively. Publication bias was assessed using the funnel plot and Egger's test.

**Results:**

A total of 32 studies were included after screening with inclusion and exclusion criteria (33481 patients). This meta-analysis found that VCD resulted in shorter time to hemostasis, ambulation, and discharge (*p* < 0.00001). In terms of vascular complication risks, VCD group might be associated with a lower risk of major complications (*p* = 0.0001), but the analysis limited to randomized controlled trials did not support this result (*p* = 0.68). There was no significant difference in total complication rates (*p* = 0.08) and bleeding-related complication rates (*p* = 0.05) between the two groups. Patient satisfaction was higher in VCD group (*p* = 0.002). Meta-regression analysis revealed no specific covariate as an influencing factor for above results (*p* > 0.05).

**Conclusions:**

Compared with MC, the use of VCDs significantly shortens the time of hemostasis and allows earlier ambulation and discharge, meanwhile without increase in vascular complications. In addition, use of VCDs achieves higher patient satisfaction and leads cost savings for patients and institutions.

## 1. Introduction

Invasive cardiac examinations and interventional procedures have become the important diagnostic and therapeutic means of cardiovascular diseases [1, 2]. More than 7 million invasive cardiac procedures are performed worldwide each year [3], and with a growing trend year by year. The modified Seldinger technique has become the standard technique to vascular puncture and sheath insertion in cardiac interventional procedures [4], but postoperative hemostasis, prolonged bed rest, and vascular-related complications remain clinical problems to be improved [5–8]. The radial approach is the preferred way of percutaneous coronary intervention (PCI) recommended by guidelines [9], which improves postoperative discomfort and complications to a certain extent. However, there are still a large number of interventional procedures requiring femoral approach, including structural cardiac intervention, catheter ablation (CA), and some PCIs under special circumstances. Effective and safe hemostasis techniques are essential to reduce the patient discomfort and the burden of complications.

Manual compression (MC) remains the current gold standard to achieve closure of percutaneous angiotomy site. However, it can be time-consuming and requires intensive compression by operator; even prolonged bed rest upon completion is required [10]. For patients, the most uncomfortable process is often not the procedure itself but the long bed rest afterwards. Therefore, vascular closure devices (VCDs) were created more than 20 years ago as an alternative to MC and have been increasingly utilized for angiotomy site closure and postoperative hemostasis. On the one side, VCDs have been reported to significantly shorten the time to hemostasis (TTH) and enable patients to ambulate at an early stage [11–13]. On the flip side, published studies have conflicting results on placement success rate and vascular complications of VCDs [14–17].

A variety of VCDs are currently available in clinical practice and can be categorized into two main groups based on closure mechanism: passive approximators, which deploy a plug, sealant, or procoagulant gel to the angiotomy site without physically occluding the angiotomy (e.g., AngioSeal, FemoSeal, Vascade, ExoSeal, SiteSeal, Celt ACD, and MynxGrip) and active approximators that physically close the angiotomy site with a suture, staple or clip (e.g., Perclose ProGlide, ProStar, and Starclose) [18, 19], indicating that the technology has changed dramatically over the past 20 years. Meta-analysis of VCDs was available as decade ago [14], but current techniques and materials have changed, and it is necessary to reevaluate the advantage of VCD and MC in clinical practice. We conducted a new systematic review and meta-analysis to analyze this issue comprehensively from multiaspect including efficacy, safety, success rates, patient satisfaction, and economic benefits.

## 2. Methods

### 2.1. Data Sources and Search Strategies

This systematic review and meta-analysis was performed referring to established methods [20]. Databases including PubMed, EMbase, Cochrane Library, and Web of Science were independently searched by two reviewers (N.P and J.G) through April 2022. Predefined search terms included “vascular closure device,” “manual compression,” “cardiovascular interventional procedure,” “cardiac intervention,” “invasive cardiac procedure,” and “cardiac catheterization” with no language restriction. Additional studies were searched from reviewing review articles and references of relevant researches manually. Any discrepancies were arbitrated by the third reviewer (R.W).

### 2.2. Inclusion and Exclusion Criteria

Inclusion criteria were applied as follows: (a) randomized-controlled trials (RCTs), observational studies, and propensity-score matched studies were included; (b) compared VCD with MC in cardiac interventional procedures; (c) contained hemostasis time parameters (efficacy) or vascular complications (safety) such as TTH, time to ambulation (TTA), access site related bleeding, hematoma, pseudoaneurysm, arteriovenous fistula, etc.; and (d) had complete and accurate outcome data. Review, case report, editorial, letter, animal study, and single cohort study were excluded. Studies were not restricted by race, sex, age, or country where the studies were conducted.

### 2.3. Data Extraction and Quality Assessment

Relevant information was obtained from the original articles and raw data files of all eligible studies and entered into a predetermined spreadsheet as follows: (a) study information (first author's name, publication year, country where the study was conducted, type of study design, operation type, sample size, VCD type, and vascular access site); (b) participant characteristics (mean age, male gender, race, and underlying disease); and (c) outcome indicators: efficacy and safety of hemostasis (TTH, TTA, time to discharge (TTD), time to discharge eligibility (TTDE), same-day discharges, hemostasis success rates, vascular complications, and patient-reported outcomes). The Cochrane Collaboration recommending tool was used for quality assessments of RCTs [21]. Non-RCTs were assessed using the Newcastle-Ottawa Scale (NOS), with scores varying from 0 to 9 depending on the quality of studies, and papers were considered high quality if they scored 7 or higher. Two reviewers preformed data extraction and quality assessment independently (N.P and J.G). Any disagreements were adjudicated by the third reviewer (R.W).

### 2.4. Statistical Analysis

Review Manager (RevMan, version 5.3) and Stata (version 12.0) were used for statistical calculations in this meta-analysis. Data of RCT studies and non-RCT were merged and analyzed separately. Statistical significance was set as *p* value of less than 0.05. Data of continuous variables represented by median and interquartile range (or max-and-min) were converted to mean and standard deviation to perform statistical analysis and data synthesis [22, 23]. Heterogeneity was assessed by calculating *I*^2^ and Cochran *Q* test, with *I*^2^ value more than 50% or *p* value of the *Q* test less than 0.1 was considered evidence of significant inconsistency [24, 25]. If heterogeneity was present, sensitivity analysis was conducted to inspect the effect of a single study on the overall risk estimate by omitting one study at a time. Meta-regression analysis was also performed to examine the sources of differences among studies. If a particular covariate had a significant effect on heterogeneity, further subgroup analysis was performed. We generated funnel plot to assess potential publication bias, and the asymmetry of the plot was evaluated by Egger's test, with *p* value of less than 0.05 indicating apparent asymmetry. Trim-and-fill analysis was used to estimate the effect of publication bias on the interpretation of the results [26].

### 2.5. Related Terms and Definitions

Due to the large number of included studies, some outcome indicators had different names or vague expressions, so we redefined the terms of important indicators and classified them consistently. TTH was defined as the time from the onset of VCD deployment or compression to complete cessation of bleeding. TTA was defined as the time from the end of procedure or leaving the cardiac catheterization laboratory to mobilization. TTD was defined as the time from the beginning of TTA to hospital discharge. Major vascular complication was defined as adverse event related to vascular puncture and closure that may cause serious consequence, require therapy, or prolong hospitalization, including large groin hematoma (usually larger than 5 cm), major bleeding that compromises hemodynamics or requiring blood transfusion, access site-related infection requiring intravenous antibiotics, retroperitoneal bleeding, and pseudoaneurysm requiring surgical repair. Minor vascular complication was defined as adverse event related to puncture and closure blood vessel that may resolve spontaneously or require no human intervention, such as small hematoma, persistent pain at vascular access site, slight bleeding of access site requiring no recompression, transient access site-related nerve injury, and pseudoaneurysm requiring no therapy. Bleeding-related complication was defined as access site bleeding, groin hematoma, and retroperitoneal bleeding. Injury-related complication was defined as tissue damage around the vascular access site, including pseudoaneurysm, arteriovenous fistula, infection, nerve injury, and pain. Related terms were used according to the definitions of previous clinical trials [27].

## 3. Results

### 3.1. Search Results

**Figure 1:**
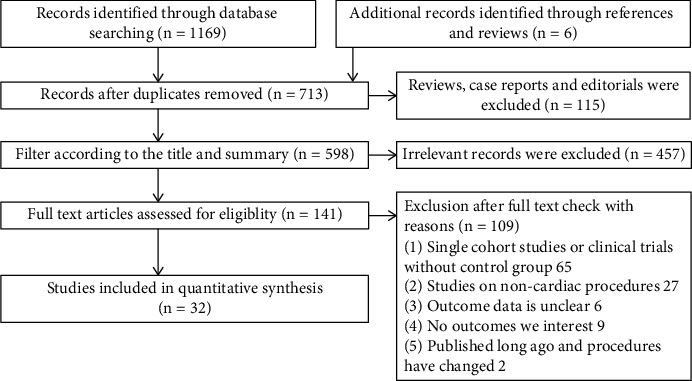
Flow diagram for study identification and inclusion.

A total of 1175 studies were initially identified through database search (1169 records) and additional manual search (6 records). After removing 462 duplicate studies, step by step screening was performed based on inclusion and exclusion criteria. Eventually, 32 studies comprising 12 RCTs [28–39], 17 observational studies [40–55], and 3 propensity-score matched studies [56–58] were included in this meta-analysis.  Figure 1 shows the flowchart of inclusions and exclusions.

### 3.2. Study Characteristics

The included studies comprised 34381 patients and were conducted in centers across the United States, Germany, China, Denmark, France, Canada, Italy, and India. The mean age of the entire cohort was 64.6 years, and participants were predominantly male (63.0%). Regarding the type of procedure, most studies were coronary angiography (CAG) and PCIs; the rest were structural cardiac procedures, CA, cardiac catheterization, etc. Regarding vascular access site, 26 studies performed procedures via femoral arteries, 4 studies via femoral veins, and 2 studies via brachial arteries. There were passive and active approximators involving 11 product types about the VCD types. The detailed characteristics of all included studies are showed in Table 1.

### 3.3. Quality Assessment

**Figure 2:**
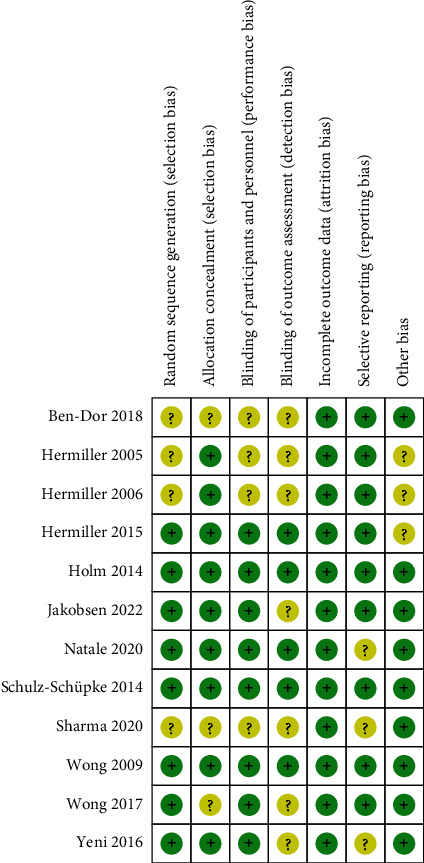
Risk of bias summary of included RCTs in the meta-analysis.

All included studies were classified as high quality according to the Cochrane Collaboration criteria or NOS.  Figure 2 and Supplementary Figure 1 show the details of quality assessment for RCTs, and results of assessment for non-RCTs is shown in Table 2.

### 3.4. Hemostasis Time Parameters

The main included clinical outcomes of hemostasis time parameters contained TTH, TTA, and TTD, and there were obvious differences in results between two groups and among studies. Notably, in terms of TTD, due to some confounding factors (e.g., delayed discharge formalities, additional examination, or consultation due to other indisposition) in included studies, TTD might not accurately reflect the efficacy of hemostasis. Therefore, the concept of time to discharge eligibility (TTDE) was introduced to reduce the error and incorporated in the subsequent quantitative synthesis on TTD.

**Figure 3:**
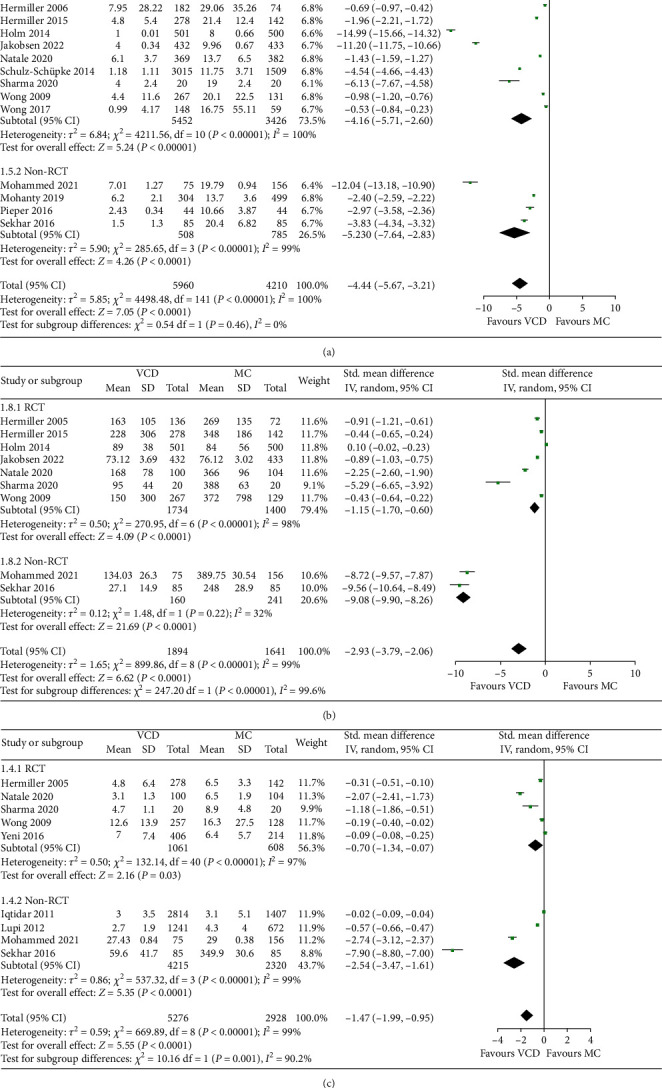
Forest plots comparing (a) TTH, (b) TTA, and (c) TTD between the VCD group and MC group.

15 studies reported the TTH, which in VCD group was significantly shorter than that in MC group (SMD: − 4.44, random-effect model, 95% CI, − 5.67 to − 3.21, *p* < 0.00001; Figure 3(a)) with high heterogeneity across studies (*I*^2^ = 100%, *p* < 0.00001 of *Q* test). 9 studies reported parameters of TTA. Similar to TTH, the result of pooled analysis suggested that use of VCD had a shorter TTA than MC (SMD: − 2.93, random-effect model, 95% CI, − 3.79 to − 2.06, *p* < 0.00001; Figure 3(b)) with high heterogeneity (*I*^2^ = 99%, *p* < 0.00001 of *Q* test). 9 studies provided related data of TTD. Data synthesis showed that VCD group had a significantly shorter length of stay (SMD: − 1.47, random-effect model, 95% CI, − 1.99 to − 0.95, *p* < 0.00001; Figure 3(c)) with high heterogeneity (*I*^2^ = 99%, *p* < 0.00001 of *Q* test). Results of RCT subgroup and non-RCT subgroup were consistent on statistical significance.

Sensitivity analysis excluding one study at a time did not find any single study significantly affecting above results and overall heterogeneity. Heterogeneity was further explored in subsequent meta-regression analysis, as described in 3.9.

**Figure 4:**
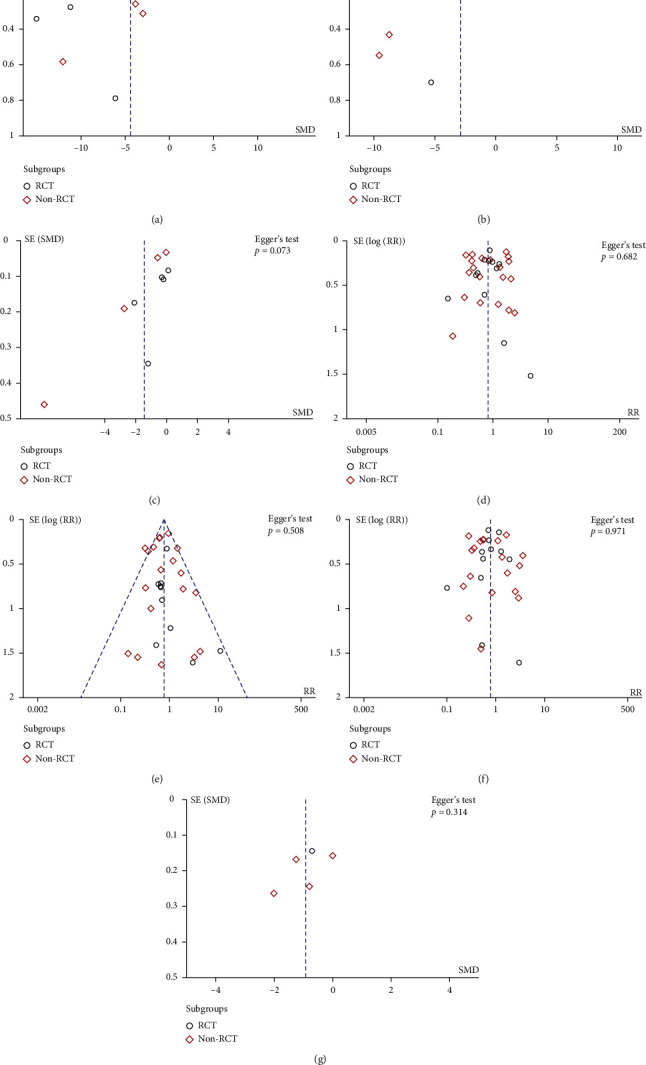
Funnel plots and Egger's test were used to assess publication bias of (a) TTH, (b) TTA, (c) TTD, (d) total vascular complication rate, (e) major vascular complication rate, (f) bleeding-related complication rate, and (g) patient-reported outcome.

No significant publication biases of TTH and TTD were observed in funnel plots and Egger's tests (Figures 4(a) and 4(c)). However, significant publication bias of TTA was revealed by funnel plot and Egger's test (*p* = 0.003, Figure 4(b)). The trim-and-fill computation was further performed to estimate the effect of publication bias on the interpretation of results. After two iterations of linear estimation and incorporating possible missing studies into the meta-analysis, the results showed no trimming was required, indicating that the impact of publication bias on the results was within an acceptable range and the result of pooled analysis was robust (Supplementary Figure 2).

### 3.5. Vascular-Related Complications

#### 3.5.1. Total Complications

**Figure 5:**
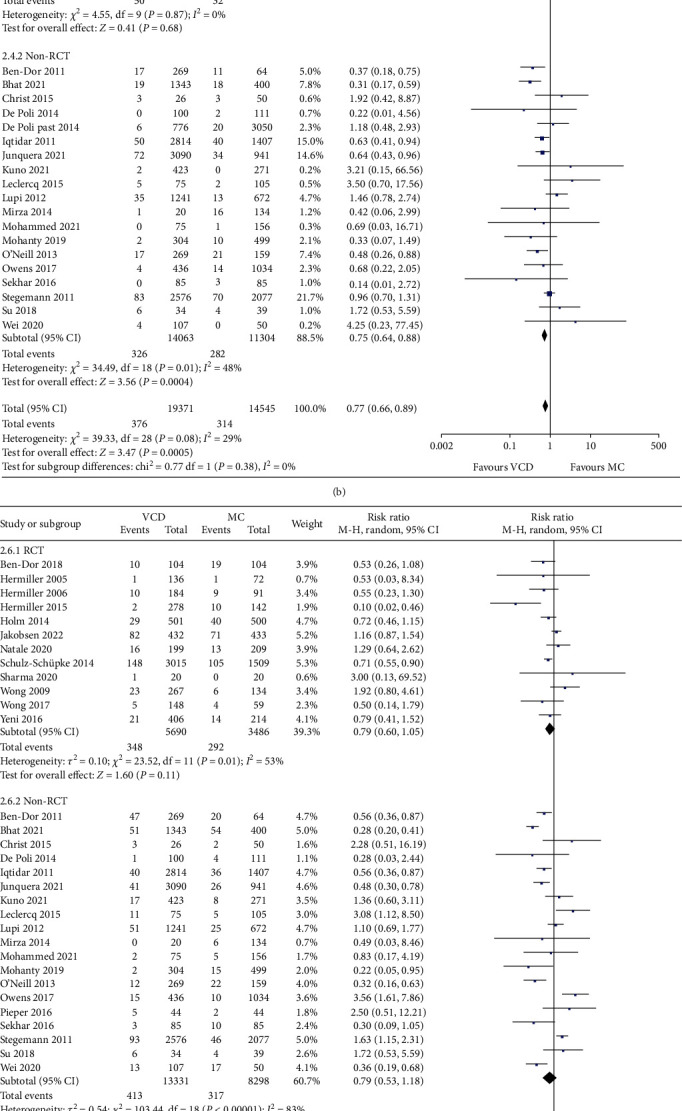
Forest plots comparing the (a) total vascular complications, (b) major vascular complications, (c) bleeding-related complications, and (d) bleeding-related complications omitting device failures between the VCD group and MC group.

All 32 studies reported vascular-related complications of cardiac interventional procedures. Of these, 13 studies favored MC, whereas 19 studies suggested that VCD could reduce complication rates. The results of quantitative synthesis showed similar total complication risks between the two methods (5.5% in VCD group and 6.0% in MC group), with no statistical significance (RR: 0.81, random-effect model, 95% CI, 0.63 to 1.02, *p* = 0.08; Figure 5(a)). And heterogeneity between studies was high (*I*^2^ = 83%, *p* < 0.00001 of *Q* test). Results were consistent in the RCT (*p* = 0.07) and non-RCT groups (*p* = 0.28).

#### 3.5.2. Major Vascular Complications

A total of 29 studies reported major vascular complications. There was no serious complication occurred in the remaining 3 studies due to the small sample sizes. The major vascular complication rate was about 1.9% of VCD group and about 2.2% of MC group according to the quantitative synthesis. The difference reached statistical significance (RR: 0.77, fixed-effect model, 95% CI, 0.66 to 0.89, *p* = 0.0005; Figure 5(b)), with low degree of heterogeneity (*I*^2^ = 15%, *p* = 0.24 of *Q* test). However, results were significantly different between RCTs and non-RCTs. The result of the RCT subgroup showed no significant difference between VCD and MC in terms of major vascular complications (*p* = 0.68), whereas the non-RCT subgroup supported the evidence that VCD effectively reduced major vascular complications (*p* = 0.0004). The most common type of major complication in both two groups (VCD and MC) was major bleeding (41.8%), followed by large hematoma (20.4%) and pseudoaneurysm (17.0%).

#### 3.5.3. Bleeding-Related Complications

Bleeding-related complications may effectively reflect the efficacy of postoperative hemostasis maintenance. A total of 28 studies provided relevant data. Similar to the result of total complications, bleeding-related complication rates were found to be lower with use of VCD compared with MC in cardiac interventional procedures, but did not reach statistical significance (RR: 0.77, random-effect model, 95% CI, 0.60 to 1.00, *p* = 0.05; Figure 5(c)). *I*^2^ was 77%, meaning a high degree of heterogeneity. However, when hemorrhagic complications caused by device failures in VCD group were removed, the result changed to favor of VCD group and reached statistical significance (RR: 0.53, random-effect model, 95% CI, 0.38 to 0.73, *p* = 0.0001; Figure 5(d)). Consistent results were observed in RCT subgroup (*p* < 0.00001) and non-RCT subgroup (*p* = 0.04), suggesting that VCDs could significantly improve hemostasis effects after successful device placements.

#### 3.5.4. Sensitivity Analysis and Publication Bias

For above results, sensitivity analyses removing one study at a time did not find significant changes on overall effect test (*p* value) and heterogeneity (*I^2^*). No significant publication biases were detected by funnel plots and Egger's tests (Figures 4(d), 4(e), and 4(f)).

### 3.6. Patient-Reported Outcomes

**Figure 6:**
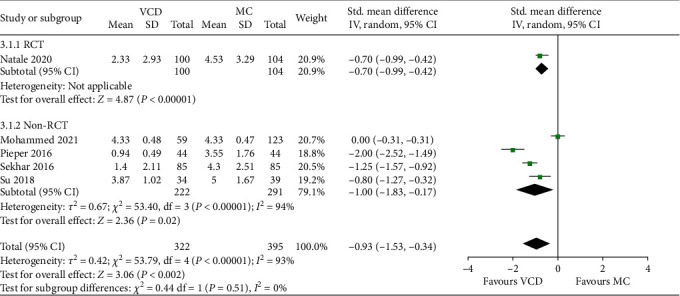
Forest plots comparing the patient-reported outcomes between the VCD group and MC group.

A total of eight studies paid additional attention to the subjective feelings of patients. Participants received questionnaires after ambulation or before discharge that comprised several items: back pain and groin pain during bed rest, discomfort in diet, urination, and defecation during bed rest, walking discomfort after ambulation, satisfaction with closure process, as well as overall satisfaction. Five of the studies quantitatively compared differences between two groups using rating scales. Because of differences in scoring rules, the data were transformed and pooled; the final results showed that patients who received VCD had higher satisfaction and less pain after procedures than who received MC (SMD: − 0.93, random-effect model, 95% CI, − 1.53 to − 0.34, *p* = 0.002; Figure 6). No significant publication bias was observed (*p* = 0.314, Figure 4(g)). Respective analysis of RCTs and non-RCTs had the consistent result. Of the three studies not included in quantitative synthesis, one observed a significant reduction in the proportion of back pain caused by prolonged bed rest in VCD group (24.3% vs 47.9%), and the other two studies showed the slight advantage of VCDs.

### 3.7. Device Failure Rates

For device failure rates of only VCD group, a total of 24 studies reported primary data, whereas the remaining studies were retrospective or propensity matching and did not report failures in original papers. Synthetic results showed that device failed at 278 of 8940 access sites for a total of 8677 participants, with a failure rate of approximately 3.1%. When device failed, either the inability to deploy the device or device deployment with inadequate hemostasis, it eventually required conversion to MC and increased the risk of bleeding-related complications.

### 3.8. Economic Benefits for Patients and Institutions

Two studies examined the costs of two closure strategies that involved passive approximator (Vascade) and active approximator (ProGlide). Both studies suggested that the use of VCDs resulted in significant cost savings for institutions and patients. Specifically, although patients had to pay for VCDs, the nursing expenses were saved due to fewer complications and shorter length of stay; meanwhile, the proportion of patients who required urinary catheter and pain medication after procedures was lower. Thus, population-level cost analysis revealed the advantages of VCDs. For example, one of the studies showed an average savings of $983.6 per patient undergoing cardiac catheterization using VCD.

### 3.9. Meta-Regression Analysis

There are some results of pooled analysis in this meta-analysis had high heterogeneity, but no significant change of heterogeneity could be observed by sensitivity analysis. Hence, meta-regression analyses were preformed to further search for the source of inconsistency between studies. Covariates included publication year, country where research was conducted, study design (RCT or observational study), operation type, VCD type (active or passive approximators), diagnosis or treatment, and vascular access site. The detailed results of the meta-regression analysis are presented in Table 3. Notably, only the analysis for total complications showed a decrease in *τ* square from 0.3168 to 0.2957, indicating that the above covariates could explain 6.7% of heterogeneity, whereas *τ* square of other indicators did not decrease. The final meta-regression results for all outcome indicators showed differences in included covariates were not the main factors affecting overall heterogeneity (*p* > 0.05).

## 4. Discussion

In this systematic review and meta-analysis, we comprehensively analyzed the performances of using VCDs versus conventional MC to close vascular access sites in cardiac interventional procedures. The main findings include the following: (1) VCDs significantly shorten the time of immediate hemostasis and postoperative bed rest, greatly increased the possibility of early discharge; (2) both showed similar results in terms of total vascular complications, but VCDs possibly reduced the risk of major complications and bleeding-related complications omitting device failures; (3) the use of VCDs increased patient satisfaction with the entire procedure; and (4) the use of VCDs contributes to cost saving for patients and hospitals.

The difference in hemostasis efficacy of the two methods is quite obvious. In most cases, complete hemostasis by VCDs takes only a few minutes, and fewer subsequent bleeding-related complications occur once the device success. Of course, VCDs have a certain failure rate, which is approximately 3.1% according to our analysis. Device failure rate has decreased with the development of technology and the operator experience, but it has not yet reached the desired perfection [41]. Another evidence of the hemostasis efficacy of VCDs is the reduction of TTA, which directly determines patient satisfaction. After successful hemostasis, conventional MC requires patients to remain on bed rest for 6-12 hours depending on the operation type [39, 50, 56]. According to previous studies, back pain, inconvenient diet, dysuria, and difficult defecation were the main causes of patient discomfort during this long period [51, 52]. Patients who received VCDs were allowed to early ambulate within 2 hours, thus avoiding these troubles. A problem with TTA is that Egger's test indicates a potential publication bias, although the bias demonstrated by the trim-and-full method does not affect the interpretation of the results. According to our analysis, the source of bias could be the study design of published papers, i.e., most of the included studies directly specify TTA in both groups without recording the actual situations.

Vascular complication is the focus of attention and the most controversial issue. Previous researches have suggested that VCDs may lead to an increase in femoral artery thrombosis, arteriovenous fistula, pseudoaneurysm, and other adverse events [29, 42, 43]. However, the results of this meta-analysis showed that VCDs did not cause additional injury and may even improve the severity of complications. Specifically, the distribution of vascular complication types was similar in both groups, whereas the major complications accounted for a relatively low proportion of the total complications in the VCD group, implying that VCDs are associated with reduced severity of adverse events, such as smaller hematoma and less groin bleeding. These minor complications are often self-healing without treatment. Our analysis confirms the safety and reliability of VCDs, and the robustness of these results is supported by the sensitivity analysis.

One notable point is that the analysis of major complications showed different results in RCTs and non-RCTs, with no significant difference between the two methods in the RCT subgroup, whereas the non-RCT subgroup favored VCD as reducing the risk of major complications. We carefully analyzed possible reasons that, first, non-RCTs might have unequal baseline patient characteristics due to study design limitations and, second, most included non-RCTs were not strictly double-blind, which might result in observer bias in assessing patients' complications. These reasons may contribute to the tendency of the results of non-RCTs to be positive. Therefore, from the perspective of evidence-based medicine, we cannot assume that VCD can reduce the risk of major complications.

Another interesting phenomenon is that, although VCDs were associated with lower bleeding-related complication rates according to this meta-analysis (4.0% vs. 5.2%), it did not reach statistical significance. One possible explanation is that VCDs indeed promote the efficiency of hemostasis, but the increased number of minor bleeding complications was driven by device failure [50]. We found evidence to support this explanation from the included studies, namely, that device failure increased the incidence of minor bleeding complications and partially offset the benefits of VCDs [35, 38, 41].

Regarding economic benefits, although there are no data that can be used for quantitative synthesis, all previous studies supported that VCDs can save costs. Notably, the cost analysis was based on the procedure success of VCDs, whereas patients would face more expensive costs once the device failed than MC. Therefore, it is important to improve device success rate and shorten the learning curve of operator in the future.

The high heterogeneity of multiple outcome indicators was observed in this meta-analysis, but neither sensitivity analysis omitting one study at a time nor meta-regression analysis found the source of inconsistency among studies. We considered that different proficiency of operators and characteristics of study population may be the reason for this result. Of course, it may also be related to the quality of included studies, that is, the accuracy and potential bias of the data. Larger real-world studies may be needed in the future to verify these conclusions.

## 5. Limitations

A limitation of this analysis is that high heterogeneity among included studies was found for most outcomes indicators. Although in most cases no factor was found to influence heterogeneity by meta-regression analysis, the effect of different baseline characteristics on outcomes cannot yet be fully assessed due to unclear reports such as race, operator experience and patient condition. Second, although included studies passed quality assessments, there were study characteristics that pose potential bias risk such as non-RCT, open-label design and related instrument manufacturer funding. Finally, duo to the lack of examination results such as ultrasound for access site, the assessment of vascular complications in some studies was based only on symptoms and patient perceptions, which may lead to potential bias.

## 6. Conclusions

The use of VCDs significantly shortens the hemostasis time and allows earlier ambulation and discharge, with the comparable safety as compared with MC. In addition, the use of VCDs achieves higher patient satisfaction and leads cost savings for patients and institutions.

## Figures and Tables

**Table 1:** Summary of included studies.

Study	Publication year	Research country	Study type	Operation type	Access site	VCD type	Sample size	Age (mean ± SD)	Male gender *n* (%)
Ben-Dor [28]	2018	USA	RCT	PCI/CAG	Femoral vein	MynxGrip	208	72.5 ± 14.2	117 (56.3)
Ben-Dor [40]	2011	USA	Retrospective study	BAV	Femoral artery	AngioSeal/Perclose/Prostar	333	81.8 ± 9.3	146 (43.8)
Bhat [41]	2021	India	Retrospective study	PCI	Femoral artery	Perclose	1743	52.1 ± 11.2	1097 (62.9)
Christ [42]	2015	Germany	Retrospective study	PCI/CAG	Femoral artery	AngioSeal	76	64.2 ± 12.8	46 (60.5)
De Poli [43]	2014	France	Retrospective study	PCI/CAG	Femoral artery	FemoSeal	211	63.2 ± 12.2	76 (76.0)
De Poli past [43]	2014	France	Retrospective study	PCI/CAG	Femoral artery	Unknown	3826	Unknown	Unknown
Hermiller [29]	2005	USA	RCT	CAG	Femoral artery	Starclose	208	61.7 ± 11.8	139 (66.8)
Hermiller [30]	2006	USA	RCT	PCI	Femoral artery	Starclose	275	62.8 ± 9.9	221 (80.4)
Hermiller [31]	2015	USA	RCT	CC	Femoral artery	Vascade	420	62.0 ± 10.9	298 (71.0)
Holm [32]	2014	Denmark	RCT	CAG	Femoral artery	FemoSeal	1001	64.8 ± 11.0	621 (62.0)
Iqtidar [56]	2011	USA	Propensity match	PCI	Femoral artery	AngioSeal/Starclose/Perclose	4221	65.4 ± 12.5	2076 (64.1)
Jakobsen [33]	2022	Denmark	RCT	CAG	Femoral artery	MynxGrip	865	66.0 ± 11.0	570 (65.9)
Junquera [57]	2021	Canada	Propensity match	TAVR	Femoral artery	AngioSeal/Perclose	4031	80.8 ± 7.8	1921 (47.7)
Kuno [58]	2021	USA	Propensity match	PCI	Femoral artery	AngioSeal/Perclose	694	66.7 ± 9.7	529 (76.2)
Leclercq [44]	2015	France	Prospective study	BAV	Femoral artery	AngioSeal	180	83.8 ± 6.8	84 (46.7)
Lupi [45]	2012	Italy	Retrospective study	PCI/CAG	Femoral artery	AngioSeal	1913	Unknown	Unknown
Mirza [46]	2014	USA	Retrospective study	CC	Brachial artery	Starclose	148	69.5 ± 8.6	79 (53.4)
Mohammed [47]	2021	USA	Prospective study	CA	Femoral vein	Perclose	231	64.9 ± 10.7	145 (62.8)
Mohanty [48]	2019	USA	Retrospective study	CA/LAAC	Femoral vein	Vascade	803	66.1 ± 10.2	538 (70.0)
Natale [34]	2020	USA	RCT	CA	Femoral vein	Vascade	204	62.5 ± 11.3	131 (64.2)
O'Neill [49]	2013	USA	Retrospective study	BAV	Femoral artery	Perclose	428	83.7 ± 8.9	194 (45.3)
Owens [50]	2017	USA	Retrospective study	CC	Femoral artery	Cardiva catalyst II	1470	63.9 ± 9.7	1419 (96.5)
Pieper [51]	2016	Germany	Prospective study	CC	Femoral artery	ExoSeal	48	62.5 ± 12.6	29 (60.4)
Schulz-Schüpke [ 35]	2014	Germany	RCT	CAG	Femoral artery	FemoSeal/ExoSeal	4524	67.0 ± 11.8	3129 (69.2)
Sekhar [52]	2016	USA	Prospective study	CC	Femoral artery	Perclose	170	59.5 ± 11.0	149 (87.6)
Sharma [36]	2020	USA	RCT	CC	Femoral artery	SiteSeal	39	60.5 ± 9.5	23 (59.0)
Stegemann [53]	2011	Germany	Retrospective study	PCI/CAG	Femoral artery	AngioSeal	4653	65.0 ± 11.6	3233 (69.5)
Su [54]	2018	China	Retrospective study	PCI	Femoral artery	AngioSeal	73	66.8 ± 12.1	52 (71.2)
Wei [55]	2020	China	Retrospective study	TBAD	Brachial artery	ExoSeal	157	57.8 ± 13.1	124 (79.0)
Wong [37]	2017	USA	RCT	PCI	Femoral artery	Celt ACD	207	67.0 ± 11.0	159 (76.8)
Wong [38]	2009	USA	RCT	PCI/CAG	Femoral artery	ExoSeal	401	62.7 ± 10.9	265 (66.1)
Yeni [39]	2016	Germany	RCT	PCI	Femoral artery	AngioSeal/Starclose	620	65.7 ± 11.1	444 (71.6)

VCD = vascular closure device; USA = the United States of America; RCT = randomized controlled trial; PCI = percutaneous coronary intervention; CAG = coronary angiography; BAV = balloon aortic valvuloplasty; CC = cardiac catheterization; TAVR = transcatheter aortic valve replacement; CA = catheter ablation; LAAC = left atrial appendage closure; TBAD = type B aortic dissection.

**Table 2:** Quality assessment of non-RCTs.

Study	Publication year	NOS score
Ben-Dor [40]	2021	8
Bhat [41]	2021	9
Christ [42]	2015	8
De Poli [43]	2014	9
De Poli past [43]	2014	7
Iqtidar [56]	2011	8
Junquera [57]	2021	7
Kuno [58]	2021	9
Leclercq [44]	2015	8
Lupi [45]	2012	7
Mirza [46]	2014	8
Mohammed [47]	2021	9
Mohanty [48]	2019	7
O'Neill [49]	2013	8
Owens [50]	2017	8
Pieper [51]	2016	8
Sekhar [52]	2016	8
Stegemann [53]	2011	7
Su [54]	2018	8
Wei [55]	2020	8

RCT = randomized controlled trial; NOS = Newcastle-Ottawa scale.

**Table 3:** Results of meta-regression analysis for outcome indicators.

Variable	Slope coefficient	Standard error	*Z* value	*p* value	95% CI	
					Lower limit	Upper limit
*Total vascular complication*						
Publication year	− 0.0038882	0.0366959	− 0.11	0.916	− 0.0796248	0.0718485
Research country	0.2122738	0.1532003	1.39	0.179	− 0.1039161	0.5284636
Study design	0.1899589	0.3187227	0.6	0.557	− 0.4678523	0.8477701
Operation type	− 0.1619395	0.170639	− 0.95	0.352	− 0.5141212	0.1902422
VCD type	− 0.0748574	0.1508855	− 0.5	0.624	− 0.3862698	0.2365551
Diagnosis or treatment	0.0898802	0.2147782	0.42	0.679	− 0.3534002	0.5331606
Vascular access site	0.0477251	0.2622489	0.18	0.857	− 0.4935301	0.5889803
*Major vascular complication*						
Publication year	0.0134347	0.0294913	0.46	0.653	− 0.0478958	0.0747652
Research country	0.1285218	0.0682783	1.88	0.074	− 0.0134707	0.2705144
Study design	0.0534072	0.1441656	0.37	0.715	− 0.2464016	0.353216
Operation type	− 0.2259866	0.1351589	− 1.67	0.109	− 0.5070649	0.0550916
VCD type	0.0388801	0.1573338	0.25	0.807	− 0.2883134	0.3660736
Diagnosis or treatment	0.2951529	0.2025009	1.46	0.160	− 0.1259707	0.7162765
Vascular access site	− 0.1544536	0.2936646	− 0.53	0.604	− 0.7651627	0.4562555
*Bleeding-related complication*						
Publication year	0.0071636	0.0433535	0.17	0.870	− 0.0825199	0.0968471
Research country	− 0.0502883	0.2334795	− 0.22	0.831	− 0.5332774	0.4327009
Study design	0.3204116	0.1969521	1.63	0.117	− 0.087015	0.7278381
Operation type	− 0.3396131	0.1908548	− 1.78	0.088	− 0.7344263	0.0552
VCD type	− 0.2991253	0.2131044	− 1.4	0.174	− 0.7399653	0.1417147
Diagnosis or treatment	0.1150813	0.2458099	0.47	0.644	− 0.3934151	0.6235778
Vascular access site	− 0.2120466	0.290403	− 0.73	0.473	− 0.8127909	0.3886977
*TTH*						
Publication year	− 0.4921285	0.3435058	− 1.43	0.195	− 1.304391	0.3201336
Research country	− 5.162946	3.383787	− 1.53	0.171	− 13.16433	2.83844
Study design	0.5445317	2.421016	0.22	0.828	− 5.180262	6.269325
Operation type	1.148615	1.776338	0.65	0.538	− 3.051757	5.348986
VCD type	− 4.169431	3.268597	− 1.28	0.243	− 11.89843	3.559573
Diagnosis or treatment	1.495201	2.149798	0.7	0.509	− 3.588264	6.578666
Vascular access site	− 0.6847343	4.187115	− 0.16	0.875	− 10.58569	9.216219
*TTA* ^∗^						
Publication year	− 0.3388484	0.2746872	− 1.23	0.343	− 1.520732	0.8430351
Research country	− 1.871418	4.513303	− 0.41	0.719	− 21.2906	17.54776
Study design	− 6.795713	1.759985	− 3.86	0.061	− 14.36832	0.7768918
Operation type	− 2.313697	2.0215	− 1.14	0.371	− 11.01151	6.384116
VCD type	− 6.795713	1.759985	− 3.86	0.061	− 14.36832	0.7768922
Diagnosis or treatment	− 2.478133	1.655155	− 1.5	0.273	− 9.599688	4.643422
Vascular access site	2.048284	2.764677	0.74	0.536	− 9.847159	13.94373
*TTD*						
Publication year	0.0616289	0.6462428	0.1	0.939	− 8.149665	8.272922
Research country	− 0.3444556	3.792939	− 0.09	0.942	− 48.53832	47.84941
Study design	− 0.1481234	1.674416	− 0.09	0.944	− 21.4236	21.12736
Operation type	− 10.58069	11.3521	− 0.93	0.522	− 154.8228	133.6614
VCD type	− 4.791256	5.455508	− 0.88	0.541	− 74.11005	64.52754
Diagnosis or treatment	− 9.694279	11.71157	− 0.83	0.560	− 158.5038	139.1153
Vascular access site	1.529658	5.465324	0.28	0.826	− 67.91387	70.97318
*Patient-reported outcome* ^∗∗^						
Publication year	− 25.04569	23.21049	− 1.08	0.476	− 319.963	269.8716
VCD type	− 38.13615	38.23929	− 1	0.501	− 524.0125	447.7402
Vascular access site	− 172.319	97.29436	− 1.77	0.327	− 1408.561	1063.923

^∗^Stata suggested that colinearity between covariate *study design* and *VCD type*. ^∗∗^Some covariates were not included in this meta-regression analysis because sensitivity analysis had been performed.

## Data Availability

All the included studies data used to support the findings of this study are included within the article and have DOI numbers in the references.

## Supplementary Materials

Supplementary 1 Risk of bias graph of included RCTs in the meta-analysis was showed in the Supplementary Figure 1.

Supplementary 2 Firm-and-full analysis of potential publication bias of TTA was showed in the Supplementary Figure 2.
