# Supplementary figures and images for: Vascular Closure Devices versus Manual Compression in Cardiac Interventional Procedures: Systematic Review and Meta-Analysis
Source: Cardiovasc Ther. 2022 Sep 9;2022:8569188. doi: 10.1155/2022/8569188 (PMC9482152; doi:10.1155/2022/8569188)

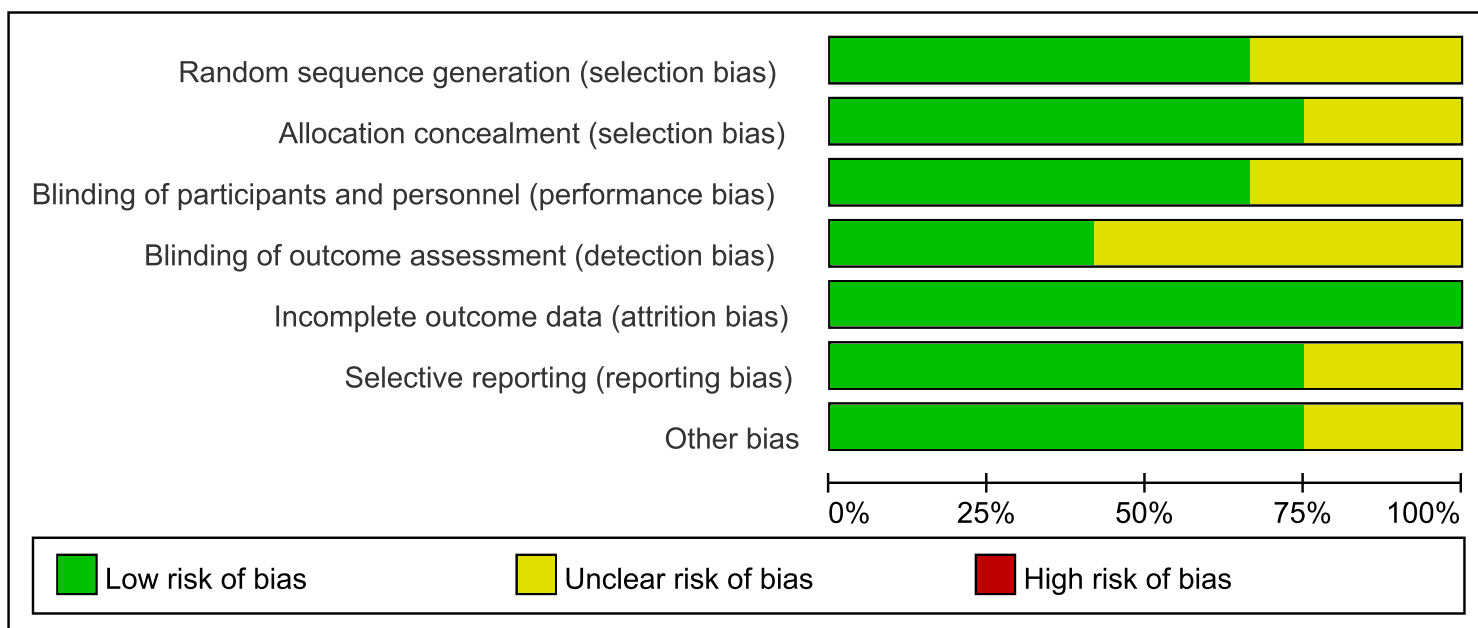

Supplement: Supplementary 1 — Risk of bias graph of included RCTs in the meta-analysis was showed in the Supplementary Figure 1. [file 8569188.f1.pdf]
