# Supplementary material for: Vascular Closure Devices versus Manual Compression in Cardiac Interventional Procedures: Systematic Review and Meta-Analysis
Source: Cardiovasc Ther. 2022 Sep 9;2022:8569188. doi: 10.1155/2022/8569188 (PMC9482152; doi:10.1155/2022/8569188)
Supplement: Supplementary 2 — Firm-and-full analysis of potential publication bias of TTA was showed in the Supplementary Figure 2. [file 8569188.f2.pdf]

Filled funnel plot with pseudo 95% confidence limits

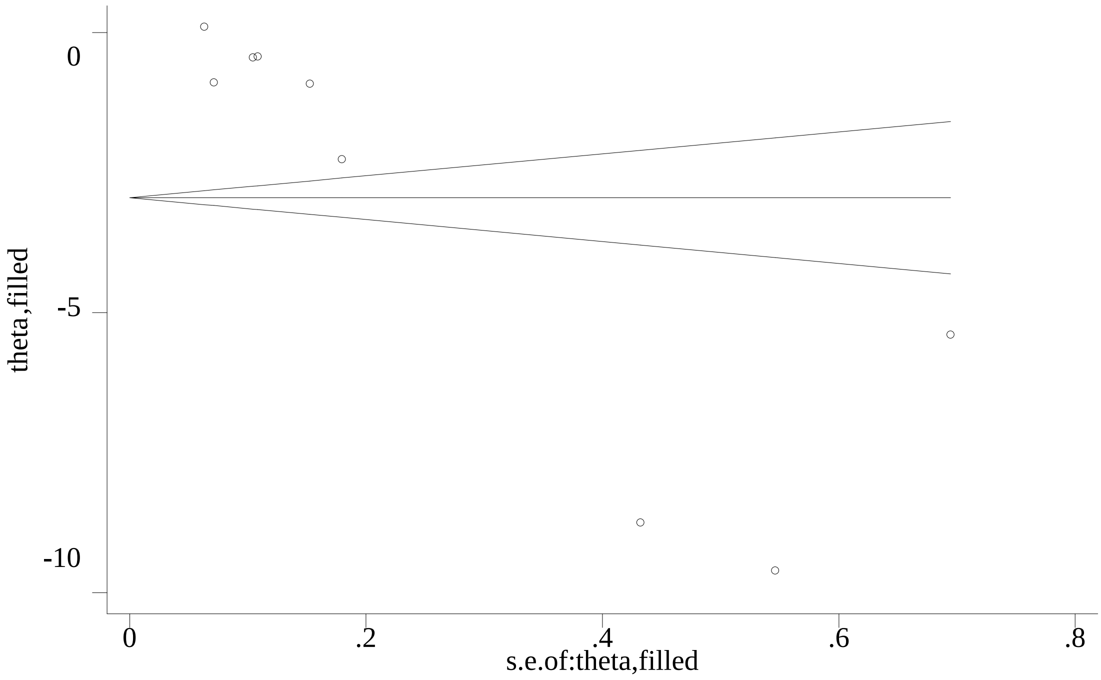

Trimming estimator: Linear
Meta-analysis type: Random-effects model

iteration	estimate	Tn	to trim	different
1	-2.947	24	0	45
2	-2.947	24	0	0

Note: no trimming performed; data unchanged
